# Analysis of multiple cytokines in aqueous humor of patients with idiopathic macular hole

**DOI:** 10.1186/s12886-020-01782-6

**Published:** 2021-01-11

**Authors:** Heping Wang, Yuqi Li, Song Han, Tongtong Niu

**Affiliations:** 1 Department of Ophthalmology, The Fourth Hospital of Shenyang, 110016 Shenyang, Liaoning People’s Republic of China; 2 University of Chinese Academy of Sciences, Beijing, People’s Republic of China; 3 Institute of Applied Ecology, Chinese Academy of Sciences, Shenyang, Liaoning People’s Republic of China

**Keywords:** Macular hole, Cytokines, Data analysis

## Abstract

**Background:**

Idiopathic macular holes are common ophthalmic manifestations with unknown pathogenesis. Thus far, there has been minimal research regarding the causes of idiopathic macular holes, especially with respect to the underlying immune mechanism. To provide clarity regarding the treatment and prognosis of idiopathic macular holes, specifically regarding the levels of cytokines in affected patients, this study examined and analyzed multiple cytokine levels in aqueous humor from patients with idiopathic macular holes.

**Methods:**

This comparative cross-sectional study included 38 patients in two groups: a cataract control group (*n* = 17) and an idiopathic macular hole group (*n* = 21). The levels of 48 cytokines in aqueous humor were detected by multiplex analysis with antibody-coupled magnetic beads. The Kolmogorov–Smirnov test was used to check whether the data were normally distributed; Student’s t-test and the Mann–Whitney U test were used to assess differences in cytokine levels between the two groups. Spearman correlation analysis was used to assess relationships among cytokine levels in the experimental group. Signaling pathways containing cytokines with significantly different expression in the experimental group were identified.

**Results:**

There were significant differences in aqueous humor cytokine levels between patients with idiopathic macular holes and patients in the cataract control group. Notably, hepatocyte growth factor (*p* = 0.0001), GM-CSF (*p* = 0.0111), and IFN-γ (*p* = 0.0120) were significantly upregulated in the experimental group, while TNF-α (*p* = 0.0032), GRO-α (*p* < 0.0001), and MIF (*p* < 0.0001) were significantly downregulated in the experimental group. Furthermore, the GM-CSF level showed significant positive correlations with levels of IL-1 (*r* = 0.67904, *p* < 0.001), IL-4 (*r* = 0.76017, *p* < 0.001), and IFN-γ (*r* = 0.59922, *p* = 0.004097) in the experimental group. Moreover, the levels of nerve growth factor and hepatocyte growth factor showed a significant positive correlation (*r* = 0.64951, *p* = 0.001441) in the experimental group.

**Conclusions:**

Patients with idiopathic macular holes showed significant variation in aqueous humor immune response after the onset of hole formation, including the recruitment of immune cells and regulation of cytokine expression. Our findings also suggest that it is not appropriate to use patients with macular holes as the control group in studies of aqueous humor cytokine levels in ophthalmic diseases.

## Background

Macular holes constitute tissue defects that extend from the retinal inner membrane to the photoreceptor layer of the macula, seriously impairing central vision in affected patients. Kuhnt first reported non-traumatic macular holes in 1900 [1]. Since then, various causes of macular holes have been recognized [2]. The overall prevalence of macular holes is 0.33%; affected patients most commonly exhibit agnogenic idiopathic macular holes (approximately 83% of patients with macular holes). Moreover, macular holes most frequently occur in healthy women aged > 50 years (mean age, 65 years; female: male ratio of 2:1); 6–28% of affected patients have bilateral macular holes [3].

Idiopathic macular holes (IMHs) have no obvious systemic or ocular local causes, and do not involve disease in the fundus itself. Current research suggests that IMHs are mainly associated with age-related degenerative changes in the vitreoretinal interface. In recent years, IMHs have become increasingly important in clinical practice because of the large number of older people in many countries, and the morbidity of this condition has gradually increased [4].

The pathogenesis of IMHs has been unclear. In 1995, Gass proposed a theory of posterior vitreous tangential traction [5]. Based on this theory, vitrectomy combined with internal limiting membrane stripping became a common surgical procedure for the treatment of IMHs, although this theory was unable to explain the molecular mechanism underlying IMH formation. Investigation of this mechanism is important for preventing the occurrence of IMHs [6].

In prior studies involving histological analysis of the internal limiting membrane in patients with IMHs, components of Müller cells, glial cells, and fibroblasts were identified [7, 8]. The present analysis of cytokines in aqueous humor was performed to investigate whether intraocular cell proliferation is mediated by inflammatory and immune cells, as well as cytokines secreted by these cells, during the occurrence, development, and repair of IMHs.

## Methods

### Participants and ethics approval

The experimental group in this study included 21 eyes of 21 patients (1 man and 20 women) with stage ≥ 3 IMHs from April 2018 to April 2019. The control group comprised 17 eyes of 17 non-diabetic patients who underwent cataract surgery at the same time, including 12 men and 5 women. The inclusion criteria in both groups were no other retinal or optic neuropathy, except diffuse retinopathy. Exclusion criteria included (1) any other eye disease (e.g., glaucoma or uveitis), (2) history of ocular surgery, and (3) history of ocular inflammation. The baseline characteristics of patients with IMHs (*n* = 21) and controls (*n* = 17) are shown in Table 1. Participants were considered to have hypertension if their blood pressure was above 140/90 mmHg or they were taking any antihypertensive medications. Participants were considered to have hypercholesterolemia if their total fasting plasma cholesterol level was > 200 mg/dl. Participants were considered to have hypertriglyceridemia if their fasting plasma triglycerides level was > 200 mg/dl. Participants were considered to be non-smokers (no smoking for at least 1 year) or current smokers. The study was approved by the Ethics Committee of the Fourth People’s Hospital of Shenyang, People’s Republic of China, and followed the tenets of the Declaration of Helsinki. All patients provided written informed consent prior to participation in the study.

**Table 1:** Baseline characteristics of patients with IMHs (*n* = 21) and patients with cataracts (*n* = 17)

Characteristics	IMH	Cataract	*P* value
Number	21	17	-
Gender			< 0.01^a^
Male (%)	1	12	
Female (%)	20	5	
Age (SD)	66.7	69.3	0.159^b^
Hypertension (%)	76	40	0.062^a^
Body mass index (SD)	29.62	27.32	0.061^b^
Smoking (%)	42	25	0.366^a^

^a^ Pearson χ^2^ test. ^b^ Student’s t-test

### Diagnosis and surgery

This study was performed using a comparative cross-sectional method at the Fourth People’s Hospital of Shenyang (People’s Republic of China). None of the patients in the IMH group had cataracts; none of the patients in the cataract group had IMHs. All patients in the IMH group underwent complete physical and ophthalmologic examination for clinical diagnosis of IMHs, including assessments of visual acuity and relative afferent pupillary defect, as well as multifocal electroretinography, optical coherence tomography, fundus examination, and fluorescent fundus angiography. During cataract surgery, limbal puncture was performed with a sterile tuberculin syringe. An undiluted aqueous sample (0.2–0.5 ml) was drawn into the syringe, then transferred to a 2-ml centrifuge tube. Immediately after the end of the operation, 0.2–0.5 ml aqueous solution was extracted by anterior chamber puncture. All samples were snap-frozen in liquid nitrogen and stored at − 80 °C until analysis.

### Measurement of aqueous cytokine levels

A Bio-Plex Pro Human Cytokine 48-plex Screening Kit (Bio-Rad, Hercules, CA, USA) was used to determine the levels of 48 human cytokines in the collected aqueous samples (Table 2).

**Table 2:** Overview of All 48 Cytokines Analyzed With a Multiplex System(Bio-Plex) From Vitreous Samples of Patients With IMHs and Cataracts(pg/ml)

	**Macular hole**									**Cataract**									
							**Range**									**Range **			**Kruskal-Wallis**
**Cytokine**	**Mean**	**Q1**	**Median**	**Q3**	**SD**	**Se**	**Min.**	**Max.**	**n**	**Mean**	**Q1**	**Median**	**Q3**	**SD**	**Se**	**Min.**	**Max.**	**n**	
IL-1β	1.4	0.7025	1.49	1.75	0.83	0.18	0.47	3.49	21	3.53	0.43	0.92	1.37	3.58	0.8	0.16	13.15	17	0.0005
IL-1rα	86.81	51.52	86.75	102.3825	48.17	10.51	24.2	213.46	21	269.63	16.55	36.06	55.14	357.75	66.43	9.86	1413.18	17	0.0005
IL-2	8.54	5.7025	8.995	11.33	4.64	1.01	1.31	21.44	20	11.83	6.41	7.1	8.11	9.15	1.7	3.13	46.4	16	0.7357
IL-4	2.45	1.48	2.74	3.12	1.13	0.25	0.91	4.71	21	5.66	0.17	1.11	1.58	5.99	1.11	0.17	19.52	17	0.0005
IL-5	1.9	0.985	2	2.3725	0.99	0.22	0.6	3.83	21	8.61	0	0	2.63	11.67	2.17	2.63	56.79	16	0.0397
IL-6	370.35	34.93	61.11	399.745	737.63	160.96	21.53	3528.35	21	650.91	52.38	116.94	802.57	878.14	163.07	12.7	4168.2	17	0.0741
IL-7	7.84	4.26	5.375	7.2325	7.43	1.62	2.44	38.97	21	13.51	1.07	2.75	6.6	15.74	2.92	1.07	73.88	15	0.6906
IL-8	41.81	16.08	19.02	24.64	61.88	13.5	10.09	265.9	21	55.36	12.47	15.62	40.61	50.77	9.43	1.21	201.79	17	0.142
IL-9	3.14	1.9225	2.63	3.55	1.65	0.36	0.74	7.02	21	8.25	2.76	3.95	4.89	8.02	1.49	0.61	38.73	17	0.2112
IL-10	6.53	4.03	6.63	8.4975	4.53	0.99	0.88	21.58	20	14.34	5.23	8.98	10.46	9.66	1.79	2.79	38.59	15	0.1682
IL-12	28.96	11.675	25.835	31.3025	29.89	6.52	0.93	141.73	21	39.95	20.99	31.43	41.36	20.88	3.88	6.99	95.89	17	0.0917
IL-13	23.45	17.74	24.585	28.51	9.65	2.11	8.15	42.51	21	32.37	7.99	12.7	16.71	26.04	4.83	0.65	93.09	16	0.6442
IL-15	29.65	16.52	21.95	39.2575	18.28	3.99	9.17	87.99	21	23.91	9.38	11.79	21.94	15.88	2.95	3.06	68.31	17	0.628
IL-17	11.51	6.1125	11.72	13.185	5.27	1.15	3.4	21.43	20	16.26	4.35	7.19	8.83	16.44	3.05	1.89	78.37	17	0.0117
Eotaxin	28.09	17.4325	27.73	32.92	12.6	2.75	11.11	56.82	21	742.75	56.39	98.2	147.42	977.47	181.51	33.98	3845.81	16	0.0005
FGF basic	16.41	12.5375	16.965	20.87	7.02	1.53	3.45	29.19	21	43.78	16.55	18.68	25.43	33.63	6.25	5.88	135.28	16	0.0066
G-CSF	44.28	16.445	36.67	54.9775	38.13	8.32	8.54	174.53	20	102.98	0	8.29	50.71	118.05	21.92	5.8	476.41	17	0.0005
GM-CSF	97.21	84.9525	114.985	134.5425	50.45	11.01	8.48	179.42	19	90.62	33.4	48.73	62.46	58.95	10.95	23.39	239.45	17	0.0005
IFN-γ	101.4	49.0075	110.175	135.87	58.53	12.77	24.47	243.5	21	382.99	13.96	30.24	80.38	445.78	82.78	3.15	1485.18	17	0.0005
IP-10	871.21	122.57	262.485	639.6125	1268.9	276.9	86.58	5425.71	21	4900.5	1715.45	1816.82	2495.55	3973.73	737.9	1074.31	17560.98	17	0.0005
MCP-1	997.93	351.0625	475.08	1509.978	1023.72	223.39	36.64	4124.45	21	707.21	161.79	273.01	495.47	756.69	140.51	90.03	3262.17	17	0.0166
MIP-1α	1.59	0.965	1.63	1.94	0.64	0.14	0.65	2.82	21	2.3	0.25	0.88	1.3	2.09	0.39	0.25	8.2	17	0.0567
PDGF-bb	31.97	5.6425	20.665	26.0175	15.37	3.35	3.07	58.17	21	73.44	0	1.72	4.16	98.19	18.23	1.72	352.91	17	0.0005
MIP-1β	34.47	30.9575	36.365	40.475	16.74	3.65	5.88	71.59	21	114.98	31.89	48.89	60.56	98.69	18.33	23.53	408.5	17	0.0005
RANTES	13.88	9.665	14.72	17.82	6.22	1.36	2.49	24.34	21	470.34	0	24.92	38.94	580	107.7	12.63	1577.14	17	0.0005
TNF-α	18.12	9.085	19.16	21.57	11.09	2.42	4.86	46.94	21	114.82	18.67	27.72	76.06	130.03	24.15	9.44	653.59	17	0.0269
VEGF	135.77	34.2875	125.765	145.1375	153.18	33.43	0.48	659.33	20	250.22	89.83	132.38	186.67	203.13	37.72	36.01	777.65	17	0.0908
IL-1α	2.4	1.3875	1.99	3.425	1.25	0.27	0.73	4.8	21	1.92	0.03	0.46	0.57	2.88	0.53	0.03	13.6	16	0.3909
IL-2Rα	152.09	109.6725	165.06	192.67	90.14	19.67	25.53	391.08	21	210.41	64.7	86.08	106.66	218.15	40.51	42.13	997.82	17	0.7558
IL-3	128.26	59.96	96.545	121.835	52.41	11.44	69.77	224.35	21	132.32	27.99	42.61	61.22	164.33	30.52	8.4	766.23	15	0.0815
IL-12p40	495.49	324.1675	383.52	520.64	283.78	61.93	188.94	1089.9	21	526.35	227.21	263.65	297.21	456.08	84.69	186.66	2223.38	17	0.0005
IL-16	216.82	95.89	192.19	270.6875	144.79	31.6	34.13	589.99	21	375.02	120.99	178.84	203.96	345.12	64.09	34.85	1527.31	17	0.0005
IL-18	9.42	5.635	7.695	11.26	5.83	1.27	1.88	22.88	21	13.45	0.83	2.27	4.81	19.67	3.65	0.83	87.95	17	0.0005
CTACK	64.1	39.92	66.44	72.26	20.38	4.45	34.7	96.62	18	113.96	50.72	65.78	65.78	85.57	15.89	32.14	386.63	17	0.0005
GROα	38.74	28.4575	37.65	41.3925	15.61	3.41	13.58	82.22	21	205.34	98.67	131.19	149.01	155.14	28.81	36.29	892.76	17	0.0912
HGF	6529.97	841.315	1546.67	6385.628	8737.89	1906.76	292.75	27226.2	21	2099.18	443.27	480.38	555.96	3885.02	721.43	162.8	20468.49	17	0.0005
IFN-α2	24.98	18.1375	27.57	32.75	9.16	2	10.21	40.7	20	35.49	20.39	23.58	26.32	21.23	3.94	16.48	106.62	17	0.1816
LIF	11.46	6.7625	9.62	14.7	6.53	1.43	4.29	28.92	21	15.93	2.09	5.65	6.53	22.79	4.23	0.72	111.33	16	0.4171
MCP-3	92.44	50.845	107.53	137.71	49.14	10.72	4.99	149.62	21	171.41	46.52	90.45	126.19	215.65	40.05	46.52	1111.53	17	0.3872
M-CSF	44.23	33.5525	38.45	46.8425	17.17	3.75	23.41	82.98	21	43.59	16.41	17.21	22.45	47.62	8.84	8.16	228.33	17	0.0089
MIF	164.45	102.4325	133.98	149.485	200.62	43.78	24.19	1061.92	19	1055.71	287.88	372.97	641.83	1102.06	204.65	138.84	4157.03	17	0.0525
MIG	365.77	76.4475	90.84	471.555	407.37	88.9	40.83	1481.8	21	882.67	64.03	84.2	109.74	2742.61	509.29	42.8	14923.23	17	0.1039
β-NGF	2.82	1.49	2.53	3.4975	1.28	0.28	0.75	4.98	20	2.21	0.64	0.86	1.07	2.9	0.54	0.21	11.79	17	0.0704
SCF	15.37	11.4	16.235	17.505	6.25	1.36	7.35	29.03	21	37.85	7.31	10.79	15.69	46.93	8.71	1.26	217.2	17	0.0005
SCGF-β	1233.46	477.64	1147.105	1780.82	747.64	163.15	161.68	2625.67	21	1914.5	474.71	539.46	837.48	1950.35	362.17	393.98	7694.8	17	0.0005
SDF-1α	1346.26	784.2975	1202	1811.13	593.03	129.41	297.58	2027.71	21	1392.22	645.87	712.17	777.16	1221.2	226.77	361.92	5179.33	17	0.5936
TNF-β	1.76	0.645	1.18	2.57	1.07	0.23	0.32	3.7	19	5.26	0	0	0	9.46	1.76	0.92	36.58	16	0.3166
TRAIL	37.01	16.1725	26.74	38.6025	29.99	6.54	5.7	102.7	21	29.39	5.73	8.15	10.51	43.72	8.12	3.24	216.77	17	0.0005

All experimental measurements were performed in accordance with the kit manufacturer’s instructions. Briefly, 50 µl of 1 × beads were added to each well and the beads were washed twice with 200 µl of wash buffer per wash. Fifty-microliter aliquots of standards, samples, and controls (i.e., negative controls that are provided with the test kit) were added to respective wells. Plates were incubated in a shaker at 850 rpm for 30 min at room temperature. Each well was then washed three times with 100 µl of wash buffer per wash. Twenty-five microliters of 1 × detection antibody was added to each well and the plates were incubated in a shaker at 850 rpm for 30 min at room temperature. Each well was then washed three times with 100 µl of wash buffer per wash. Fifty microliters of 1 × streptavidin-PE was added to each well and the plates were incubated in a shaker at 850 rpm for 10 min at room temperature. Each well was then washed three times with 100 µl of wash buffer. Samples were resuspended in 125 µl of assay buffer and the plates were incubated in a shaker at 850 rpm for 30 s at room temperature.

The measurements were performed in accordance with the kit manufacturer’s instructions and data were acquired using the Bio-Plex TM 200 system, software version 6.0 (Bio-Rad). The standard curve for each cytokine was generated using the reference set of cytokine concentrations provided with the kit; sample concentrations of each cytokine were calculated using the multi-parameter standard curve. If a sample concentration was above or below the detection limit, it was considered and outlier and removed from analysis.

### Statistical analysis

Data are shown as mean ± standard deviation, range, median, and IQR. Statistical analysis was performed using R, version 3.6.1 (R Foundation for Statistical Computing, Vienna, Austria). The Kolmogorov–Smirnov test was used to determine whether the data were normally distributed. The Pearson χ^2^ test was used for comparisons of qualitative variables. Student’s t-test and the Mann–Whitney U test were used for comparisons of quantitative variables between two groups. Spearman correlation analysis was used to assess relationships among cytokine levels in the experimental group. *p*-values < 0.05 were considered statistically significant. Pathway enrichment analysis (http://metascape.org) was used to identify major signaling pathways that contained cytokines with significantly altered expression between groups.

## Results

### Cytokine levels in aqueous humor

**Fig. 1:**
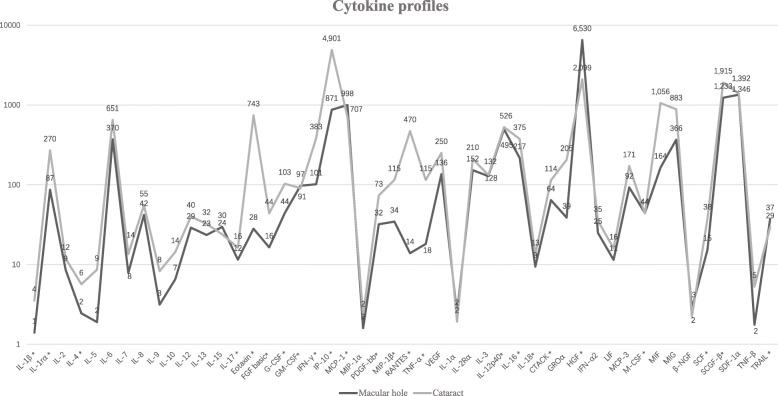
Comparison of cytokine levels between idiopathic macular hole and cataract groups using a logarithmic scale. Significant differences (*p* < 0.05) are marked with asterisks. Mean cytokine levels (pg/ml) in the idiopathic macular hole and cataract groups are shown. The *p* value is obtained by Mann–Whitney U test

**Fig. 2:**
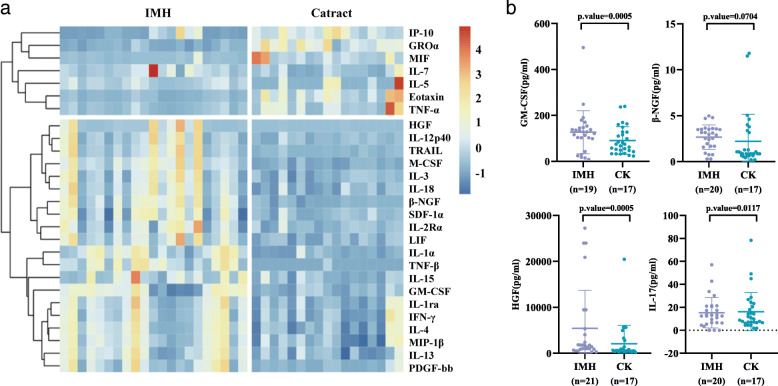
Differences in cytokine levels between idiopathic macular hole and cataract groups. **a** Differences in cytokine levels between patients with idiopathic macular holes and patients with cataracts, shown using standardized cytokine data in heat map format. The results indicated that the idiopathic macular hole and cataract groups formed two distinct clusters. **b** Quantitative differences between the two groups in levels of cytokines that may play an important role in idiopathic macular holes. The value represents the mean ±SD of cytokine concentration in cataract or IMH patients. The *p* value is obtained by Mann–Whitney U test

Levels of cytokines in aqueous humor samples were compared between the IMH and control groups (Table 2). Figure 1 shows a visual comparison of all tested cytokines between the two groups. Twenty-seven cytokines with significant differences in levels are shown in Fig. 2a; of these 27, 20 were significantly upregulated and seven were significantly downregulated. Significantly upregulated cytokines included IL-1rα (*p* = 0.0038), IL-4 (*p* = 0.0024), IL-13 (*p* = 0.0017), IL-15 (*p* = 0.0066), IL-1α (*p* = 0.0027), IL-2Rα (*p* = 0.0287), IL-3 (*p* = 0.0014), IL-12p40 (*p* = 0.0028), IL-18 (*p* = 0.0003), hepatocyte growth factor (HGF) (*p* = 0.0001), GM-CSF (*p* = 0.0111), IFN-γ (*p* = 0.0120), MIP-1α (*p* = 0.0030), PDGF-bb (*p* = 0.0027), LIF (*p* = 0.0006), M-CSF (*p* < 0.0001), β-NGF (*p* = 0.0004), SDF-1α (*p* = 0.0130), TNF-β (*p* < 0.0001), and TRAIL (*p* = 0.0001). Significantly downregulated cytokines included IL-5 (*p* = 0.0208), eotaxin (*p* < 0.0001), IP-10 (*p* = 0.0001), TNF-α (*p* = 0.0032), GROα (*p* < 0.0001), IL-7 (*p* = 0.0367), and MIF (*p* < 0.0001). Cytokines that were significantly upregulated and might be closely related to IMHs are shown in Fig. 2b.

### Relationships among cytokine levels

Table 3 shows the relationships among cytokines that were significantly different between the IMH and control groups. Notably, the level of GM-CSF was significantly positively correlated with the levels of inflammation-related cytokines including IL-1 (*r* = 0.67904, *p* < 0.001), IL-4 (*r* = 0.76017, *p* < 0.001), and IFN-γ (*r* = 0.59922, *p* = 0.004097) both. In addition, the level of neuronal and tissue repair-related nerve cell growth factor (NGF) was significantly positively correlated with the level of HGF (*r* = 0.64951, *p* = 0.001441).

**Table 3:** Correlation between cytokines with significant differences between the two groups

	IP-10	GROα	MIF	IL-7	IL-5	Eotaxin	TNF-α	HGF	IL-12(P40)	TRAIL	M-CSF	IL-3	IL-18	β-NGF	SDF-1α	IL-2ra	LIF	IL-1α	TNF-β	IL-15	GM-CSF	IL-1ra	IFN-γ	IL-4	MIP-1α	IL-13	PDGF-bb
IP-10	1.00000				-0.50895		-0.54966	0.64779	######	0.51823	0.50977	0.50880									-0.65647	-0.60963	-0.48861	-0.65604	-0.56409	-0.52115	
GROα		1.00000																0.64821	0.65471	0.53190	0.45443						
MIF			1.00000						######	0.64712	0.62561	0.66710	0.68035	0.67253	0.59550	0.52721	0.59694										
IL-7				1.00000					######				0.45247		0.44937												
IL-5	0.01846				1.00000	0.91010	0.95765														0.63846	0.96583	0.96909	0.94141	0.95607	0.96648	0.88838
Eotaxin					0.00000	1.00000	0.91851														0.55487	0.87919	0.92348	0.84756	0.90134	0.83230	0.81189
TNF-α	0.00985				0.00000	0.00000	1.00000														0.65516	0.96907	0.98991	0.96743	0.95474	0.93781	0.92619
HGF	0.00150							1.00000	######	0.78502	0.71466	0.76662	0.68990	0.64951	0.57543	0.54137	0.46450	-0.70163			-0.67773						
IL-12(P40)	0.02198		0.00141	0.02700				0.00009	######	0.98176	0.89577	0.96023	0.94267	0.93746	0.90518	0.73941	0.71726										
TRAIL	0.01610		0.00152					0.00003	######	1.00000	0.92704	0.97262	0.93094	0.94267	0.88498	0.79544	0.79153										
M-CSF	0.01824		0.00242					0.00027	######	0.00000	1.00000	0.89439	0.84756	0.89577	0.85174	0.72638	0.75505										
IL-3	0.01850		0.00096					0.00005	######	0.00000	0.00000	1.00000	0.96871	0.95763	0.90968	0.80183	0.78292										
IL-18			0.00069	0.03944				0.00054	######	0.00000	0.00000	0.00000	1.00000	0.96482	0.94493	0.72638	0.74202										
β-NGF			0.00084					0.00144	######	0.00000	0.00000	0.00000	0.00000	1.00000	0.97100	0.76808	0.82671		0.44473								
SDF-1α			0.00440	0.04099				0.00635	######	0.00000	0.00000	0.00000	0.00000	0.00000	1.00000	0.67775	0.72597										
IL-2ra			0.01405					0.01126	######	0.00002	0.00019	0.00001	0.00019	0.00005	0.00074	1.00000	0.93225										
LIF			0.00428					0.03389	######	0.00002	0.00008	0.00003	0.00012	0.00000	0.00019	0.00000	1.00000		0.53164								
IL-1α		0.00148						0.00039										1.00000	0.70166		0.67904	0.47135		0.43439	0.45898		
TNF-β		0.00128												0.04338			0.03513	0.00039	1.00000								
IL-15		0.01307																		1.00000							
GM-CSF	0.00123	0.03850			0.00184	0.00903	0.00127	0.00074										0.00071			1.00000	0.75797	0.59922	0.76017	0.67339	0.67209	0.53638
IL-1ra	0.00335				0.00000	0.00000	0.00000											0.03101			0.00007	1.00000	0.95706	0.97690	0.95576	0.95185	0.87309
IFN-γ	0.02461				0.00000	0.00000	0.00000														0.00410	0.00000	1.00000	0.94761	0.96487	0.94144	0.94291
IL-4	0.00124				0.00000	0.00000	0.00000											0.04911			0.00006	0.00000	0.00000	1.00000	0.95021	0.95151	0.88675
MIP-1α	0.00773				0.00000	0.00000	0.00000											0.03635			0.00082	0.00000	0.00000	0.00000	1.00000	0.90761	0.89005
IL-13	0.01541				0.00000	0.00000	0.00000														0.00085	0.00000	0.00000	0.00000	0.00000	1.00000	0.88353
PDGF-bb					0.00000	0.00001	0.00000														0.01219	0.00000	0.00000	0.00000	0.00000	0.00000	1.00000
The lower left part of the figure is a *p*-value and the upper-right part is an *r*-value																											

### Exploration and analysis of cytokine function

**Fig. 3:**
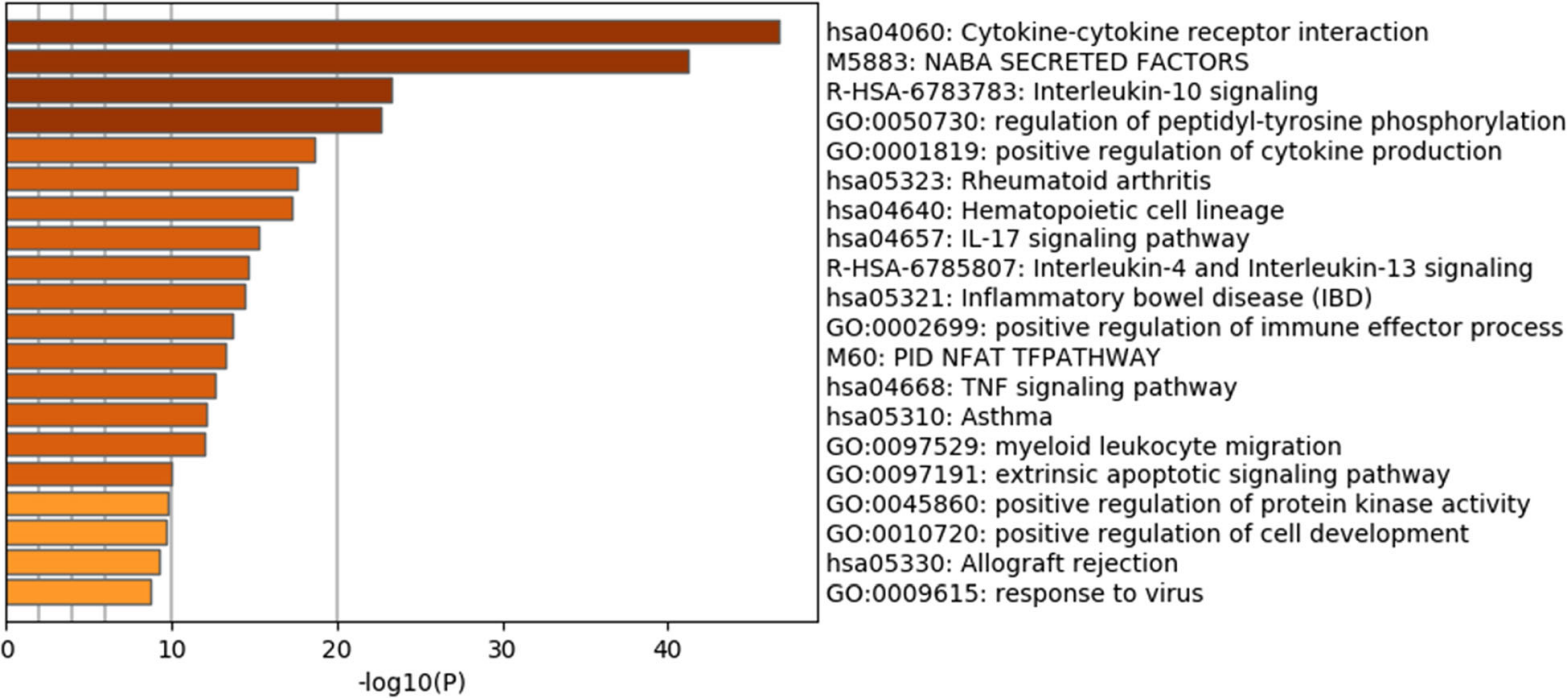
Signal pathway analysis of cytokines with significant differences in levels between the two groups. A greater value on the horizontal axis indicates greater magnitude of the between-group difference in cytokine levels involved in this pathway

Figure 3 shows the results of pathway enrichment analysis (http://metascape.org) involving cytokines that were significantly different between the IMH and control groups. The greatest proportion of cytokines was involved in the cytokine–cytokine receptor pathway, which is implicated in inflammatory responses. This includes both members of pro-inflammatory signaling pathways (e.g., IL-17, IL-14, and IL-13) and members of anti-inflammatory signaling pathways (e.g., IL-10). Moreover, the cytokine levels in hematopoietic lineage signaling pathways associated with tissue repair exhibited significant differences between groups.

## Discussion

IMH etiology or stage of development is generally considered to be unrelated to ocular inflammation; thus, affected patients are often included as a control group in analyses of inflammation-related ocular diseases [9]. However, after onset of IMHs, tissue damage and reparation often lead to local inflammatory reactions, which affect cytokine levels in the aqueous humor. The degree of ocular inflammatory response and changes in cytokine levels in the aqueous humor provide useful information for the treatment, prognosis, and diagnosis of IMH.

In the normal retina, Müller cells penetrate the neurosensory layer; the fibers of these cells extend from the outer membrane to the inner limiting membrane, thereby providing retinal support and nutrient metabolism. After IMH formation, the retina undergoes a process called reactive gliosis [10]. This event is triggered by penetration damage involving activated astrocytes. The activation of intermediate filament proteins (i.e., glial fibrillary acidic protein and vimentin) is critical for the formation of glial scars [11, 12].

In the retina, Müller cells are the major glial cell type involved in reactive scar formation following disease-induced retinal degeneration [13]. To restore nutrients, Müller cells and other glial cells in the retina may migrate to the outer surface of the inner limiting membrane in response to various inflammatory factors. Some researchers have proposed that proliferation and contraction of these cells in the inner limiting membrane results in IMH progression. After IMH formation, lymphocytes can destroy the blood–retina barrier and migrate into the vitreous cavity; here, lymphocytes make contact with transitional pigment cells and glial cells, secrete cytokines, promote an inflammatory response, and enhance macrophage-mediated phagocytosis [14]. In addition, lymphocytes in the vitreous cavity can promote the transformation of glial cells into fibroblasts and accelerate the repair of tissue hyperplasia [15].

Immune system-mediated inflammatory responses are known to play important roles in injury-induced neurological damage and tissue repair. The inflammatory response during trauma is mainly mediated by innate immune cells including microglia and astrocytes, which play important roles. However, the recruitment of these innate immune cells leads to some recruitment of T cells and other immune cells and subsequently induces apoptosis and damage repair [16].

The results of this study showed significant upregulation of GM-CSF, which is an important component of microglial products. Microglia represent a type of macrophage; thus, they can phagocytose damaged cell debris and release various cytokines. In IMHs, the microglia in the damaged macular area are rapidly activated, thereby causing microglia morphology to transform into an “ameboid-like” state and migrate to the site of injury. This is followed by the release of various cytokines including NGF, IL-4 (a protective cytokine), IL-10 (an immunosuppressive cytokine), as well as NO and ROS; pro-inflammatory cytokines are also produced (e.g., IL-1, IL-6, and IFN-γ) [17]. These cytokines might accumulate in the aqueous humor, as shown in our study. The upregulation of GM-CSF, IL-1, IL-4, and IFN-γ indicates that microglia play an important role in the immune response during IMH formation and progression.

Previous studies of cytokine levels in the vitreous of patients with ERM and patients with IMHs revealed no significant difference in GM-CSF levels between the two groups. Conversely, we found that the GM-CSF level was significantly upregulated in patients with IMHs, which implies that the use of patients with IMHs as the control group may lead to inaccurate results [18].

The significant downregulation of MIF level in the aqueous extract of patients with IMHs suggests that monocytes play important roles in the activation and development of Müller cells after the onset of IMH formation. The significant upregulation of IL-3, M-CSF, GM-CSF, and IFN-γ levels supported the notion that monocytes play important roles in IMHs. Notably, the MIF level was negatively correlated with the LIF level; LIF is an important neurogenic factor that could promote the differentiation of glial cells. LIF is mainly secreted by macrophages, which implies that macrophages play an important role in the body’s self-repair process after IMH onset.

Microglia activation and production of IL-1 cause activation of astrocytes and Müller cells. During the adaptive nerve injury in patients with IMHs, Müller cells can produce cytokines (e.g., NGF and HGF) to promote neuron survival. In this study, both NGF and HGF levels were significantly upregulated in comparison with the control group, and the NGF level was associated with the HGF level. Because HGF and NGF both play important roles in the treatment of ocular nerve injury diseases (e.g., corneal damage and glaucoma), we hypothesized that HGF and NGF also play important roles in tissue repair during the prophase and postoperative recovery phase of IMHs [19].

In this study, a multiplex analysis with antibody-coupled magnetic beads was used to detect changes in cytokine levels in aqueous humor samples from patients with IMHs. Compared with the traditional ELISA method, this method can detect a wider variety of cytokines with lower sample volume. Moreover, the method can simultaneously detect 48 cytokines in a single experiment, thus avoiding instability related to batch effects [20].

## Conclusions

This study demonstrated significant changes in the levels of various cytokines in the aqueous humor between patients with IMHs and patients with cataracts, presumably in relation to the roles of microglia and astrocytes in the pathogenesis and repair of IMHs. In addition, HGF and NGF were significantly upregulated, as were other cytokines that promote nerve growth and survival; these findings can aid in the treatment and prognosis of IMHs. Notably, patients with IMH are commonly used as the control group in research regarding aqueous humor cytokine levels in ophthalmic diseases. The results of this study suggest that the levels of multiple cytokines in the aqueous humor change considerably due to the inflammatory response in patients with IMHs. Therefore, it may not be appropriate to use patients with IMHs as a control group in studies of aqueous humor cytokine levels.

## Data Availability

The datasets generated and analyzed during the current study are not publicly available due Follow-up research needs but are available from the corresponding author on reasonable request.

## Abbreviation

IMH: Idiopathic macular hole
